# Double-Blind, Multicenter, Prospective Randomized Study of Trospectomycin Vs. Clindamycin, Both With Aztreonam, in Non-Community Acquired Obstetric and Gynecologic Infections

**DOI:** 10.1155/S1064744997000483

**Published:** 1997

**Authors:** Ashwin Chatwani, Mark Martens, Jorge Blanco, Stanley Gall, Kira Przybylko, Charles P. Wajszczuk, Dana Nickens

**Affiliations:** 1 Department of Obstetrics and Gynecology Temple University School of Medicine Philadelphia PA USA; 2 Department of Obstetrics and Gynecology Hennepin County Medical Center Minneapolis MN USA; 3 Department of Obstetrics and Gynecology LBJ Hospital Houston TX USA; 4 Department of Obstetrics and Gynecology University of Louisville Louisville KY USA; 5 Section of Antimicrobial Research Pharmacia & Upjohn, Inc. Kalamazoo MI USA; 6 Section of Medical Statistics Pharmacia & Upjohn, Inc. Kalamazoo MI USA; 7 Department of Obstetrics and Gynecology RS, 3401 N. Broad Street, 70PB Philadelphia PA 19140 USA

## Abstract

*Objective:* The purpose of this study was to compare the clinical efficacy and safety of trospectomycin sulfate with that of clindamycin phosphate, both with aztreonam, for the treatment of obstetric and gynecologic infections.

*Methods:* In a double-blind, multicenter, prospective randomized study, 579 patients with either endometritis following cesarean delivery or pelvic cellulitis following hysterectomy were enrolled and received medication. Administered was either trospectomycin sulfate 500 mg IV every 8 h or clindamycin phosphate 900 mg IV every 8 h in a 1:1 randomization ratio. Both groups of patients received aztreonam 1 g IV every 8 h. The patients were followed for clinical responses and side effects.

*Results:* The cure rate for the trospectomycin sulfate arm was 91.8% and for clindamycin phosphate arm it was 88.4% (*P* = 0.218). The adverse events were similar in both groups.

*Conclusions:* Trospectomycin was as effective as clindamycin, when both were combined with aztreonam, in treatment of obstetric and gynecologic infections.


Infectious Diseases in Obstetrics and Gynecology 5:280–285 (1997)
© 1998 Wiley-Liss, Inc.

### Double-Blind, Multicenter, Prospective Randomized Study of Trospectomycin Vs. Clindamycin, Both With Aztreonam, in Non-Community Acquired Obstetric and Gynecologic Infections

Ashwin Chatwani,[1*] Mark Martens,[2] Jorge Blanco,[3] Stanley Gall,[4] Kira Przybylko,[1] Charles P. Wajszczuk,[5] and Dana Nickens[6]

[1]*Department of Obstetrics and Gynecology, Temple University School of Medicine, Philadelphia, PA*
[2]*Department of Obstetrics and Gynecology, Hennepin County Medical Center, Minneapolis, MN*
[3]*Department of Obstetrics and Gynecology, LBJ Hospital, Houston, TX*
[4]*Department of Obstetrics and Gynecology, University of Louisville, Louisville, KY*
[5]*Section of Antimicrobial Research, Pharmacia & Upjohn, Inc., Kalamazoo, MI*
[6]*Section of Medical Statistics, Pharmacia & Upjohn, Inc., Kalamazoo, MI*

### Abstract

***Objective:*** The purpose of this study was to compare the clinical efficacy and safety of trospectomycin sulfate with that of clindamycin phosphate, both with aztreonam, for the treatment of obstetric and gynecologic infections.

***Methods:*** In a double-blind, multicenter, prospective randomized study, 579 patients with either endometritis following cesarean delivery or pelvic cellulitis following hysterectomy were enrolled and received medication. Administered was either trospectomycin sulfate 500 mg IV every 8 h or clindamycin phosphate 900 mg IV every 8 h in a 1:1 randomization ratio. Both groups of patients received aztreonam 1 g IV every 8 h. The patients were followed for clinical responses and side effects.

***Results:*** The cure rate for the trospectomycin sulfate arm was 91.8% and for clindamycin phosphate arm it was 88.4% ($P = 0.218$). The adverse events were similar in both groups.

***Conclusions:*** Trospectomycin was as effective as clindamycin, when both were combined with aztreonam, in treatment of obstetric and gynecologic infections. Infect. Dis. Obstet. Gynecol. 5: 280–285, 1997. © 1998 Wiley-Liss, Inc.

Key words
trospectomycin; clindamycin; aztreonam

Female pelvic infections that occur following obstetric and gynecologic procedures are often polymicrobial, involving both aerobic and anaerobic bacteria.[1–4] Isolated from these patients are bacteria which are similar to those isolated from the vagina and endocervix of healthy women. This observation implies that infections following obstetric and gynecologic procedures are frequently from ascent of endogenous bacteria of the vaginal flora.

A major cause of postpartum morbidity and mortality is endometritis. This is especially true in patients who have undergone cesarean delivery.[5,6] The incidence of endometritis following cesarean delivery ranges from 8 to 55%,[6–8] despite the use of perioperative prophylactic antibiotics.

Hysterectomy is one of the most common surgical procedures in the United States. Prior to widespread use of antimicrobial prophylaxis,[9] the

*Correspondence to: Dr. Ashwin Chatwani, Department of Obstetrics and Gynecology, RS, 3401 N. Broad Street, 70PB, Philadelphia, PA 19140.

Received 13 May 1997
Accepted 29 August 1997

incidence of infection following hysterectomy was 60% and included cuff cellulitis and pelvic cellulitis. The latter is observed during the immediate postoperative period, while cuff cellulitis often presents after discharge from the hospital.

The commonly isolated aerobic organisms in infections that occur after obstetric and gynecologic procedures are gram-negative facultative bacteria, especially *Escherichia coli,* hemolytic and nonhemolytic streptococcus, *Klebsiella pneumoniae,* and *Proteus* spp. The anaerobic bacteria that are frequently isolated include *Peptostreptococcus, Peptococcus,* and *Bacteroides* spp.

Parenteral antibiotic therapy is the mainstay of therapy of pelvic infections following obstetric and gynecologic procedures. Initial therapy for such infection consists of a broad-spectrum antibiotic coverage with activity against most of the *Bacteroides* spp., as well as gram-positive and gram-negative aerobes. In many clinical trials, the combination therapy with clindamycin and gentamicin has been considered the standard for comparison of the treatment of genital tract infections.

The incidence of nausea, vomiting, and diarrhea with clindamycin therapy ranges from 2 to 6%.[10] The more serious gastrointestinal problem, pseudomembranous colitis (PMC), may be greater with clindamycin than with other antibiotics. The ranges are highly variable, with a reasonable estimate being 1 in 10,000.[11]

Trospectomycin sulfate is a novel broad-spectrum aminocyclitol antibiotic with activity against a majority of aerobes, anaerobes, *Neisseria gonorrhoeae,* and *Chlamydia trachomatis.*[12–14] Trospectomycin binds to the 30-s ribosomal subunit of bacteria, thus inhibiting protein synthesis. The potential utility of this antibiotic is in the treatment of gonorrhea, gynecologic/pelvic infections, including pelvic inflammatory disease and endometritis, pneumonia, and intra-abdominal infections caused by anaerobes. In in vitro studies, trospectomycin was shown to have achievable minimal inhibitory concentrations (MICs) against a broad spectrum of pathogenic species including *Streptococcus, Haemophilus, Neisseria, Peptococcus, Peptostreptococcus, Bacteroides, Chlamydia,* and *Mycoplasma.* Reduced activity has been observed against *Enterobacteriaceae.* Trospectomycin is not active against *Pseudomonas* spp.

Clindamycin has long been one of the antibiotics of choice in the treatment of obstetric and gynecologic infections. Therefore, it is possible that resistant strains of pathogens may emerge. Because of the adverse effects and the possibility of the development of drug-resistant organisms, there is a need for a new antibiotic with a wide range of aerobic and anaerobic coverage.

The objectives of the study were to compare the safety and efficacy of trospectomycin sulfate with aztreonam vs. clindamycin phosphate with aztreonam in the treatment of infection following obstetric and gynecologic procedures.

### SUBJECTS AND METHODS

### Patients

The patients eligible for enrollment were hospitalized women, 16 years of age or older, with a presumptive diagnosis of pelvic cellulitis following hysterectomy or endometritis following cesarean delivery. The diagnosis of pelvic cellulitis was based on abdominal tenderness with or without rebound tenderness, parametrial tenderness on vaginal examination, and oral temperature ⩾38.3°C after the first 24-h period. The admission white cell count needed to be over 10,000/mm$^3$. For a diagnosis of endometritis, in addition to elevated temperature and white cell count requirements, fundal tenderness on abdominal examination along with parametrial tenderness and purulent lochia on vaginal examination needed to be present. Patients with symptoms suggesting other foci of infection, e.g., urinary tract or pulmonary system, were excluded from the study, as were patients who had received non-prophylactic antibiotics, within 14 days of recruitment in the study. Patients were also excluded if they had a history of hypersensitivity to trospectomycin, clindamycin, aztreonam, or spectinomycin, breast-feeding, myasthenia gravis, liver or kidney dysfunction, antibiotic-associated colitis, immunologic deficiency, and neoplastic diseases.

### Study Design

This was a phase III, comparative, double-blind, randomized study of trospectomycin sulfate and clindamycin phosphate, both with aztreonam, in the treatment of pelvic infection following obstetric and gynecologic procedures. The institutional review boards of the 18 participating centers approved the clinical protocol for this study. The centers were chosen from geographic areas so as to obtain a heterogeneous group of patients. The pharmaceutical sponsor provided a simple computer-generated randomization table for each site. A 1:1 allocation of screened patients to trospectomycin vs. clindamycin was used.

After each patient had given written informed consent, a physical examination was performed and the following laboratory studies were conducted: urinalysis, complete blood count (CBC) with differential, prothrombin time, direct Coomb's test, alanine aminotransferase, aspartate aminotransferase, lactic dehydrogenase, alkaline phosphatase, total bilirubin, blood urea nitrogen, and serum creatinine. Cultures were obtained from the urine, uterus, or vaginal cuff, and, if indicated, blood, prior to treatment. The indications for blood culture were left to the discretion of each individual investigator. Uterine cultures were obtained by transcervical protected brush or endometrial aspiration curette. Cultures were obtained for facultative aerobes and anaerobes. Standard culture media and culture techniques were utilized for aerobic and anaerobic bacteria. Organism susceptibility was based on either MIC or inhibition zone. If both were recorded, MIC was used. The susceptibility breakpoints for all antibiotics were established before the start of the study. An MIC for trospectomycin was established at ≤16 μg/ml, for clindamycin at ≤1.6 μg/ml, and for aztreonam at ≤8 μg/ml. An inhibitory zone diameter for trospectomycin and clindamycin was established at ≥17 mm and for aztreonam at ≥22 mm.

The treatment regimen consisted of IV therapy with 500 mg trospectomycin or 900 mg clindamycin as a 30-min infusion every 8 h. Both groups of patients received 1 g IV aztreonam every 8 h. The study medication was continued for 24 h following the resolution of the signs and symptoms of pelvic infection. No discharge antibiotics were given to either group.

### Patient Evaluation

Over the course of treatment, the patients were observed regarding their clinical status and potential adverse reactions. Specific questions were asked daily regarding any local discomfort at the IV site. Other factors included oral temperature evaluations every 4–6 h. The blood culture was repeated daily for a patient with a positive culture before treatment until it became negative. All clinical laboratory tests were repeated every 3 days while the patient was hospitalized, and again on the day of discharge.

The disappearance of all signs and symptoms of endometritis or pelvic cellulitis defined a satisfactory clinical response to treatment. A clinical failure was defined as a temperature of ≥38°C on one or more occasions in a patient who had received antibiotics for a minimum of 48 h, along with persistence or worsening of the signs and symptoms of endometritis or pelvic cellulitis that required changing the antimicrobial agents.

### Statistical Evaluation

For continuous variables, e.g., age, the treatment differences were tested using a one-way analysis of variance (ANOVA) model, with treatment as the factor. Categorical variables, e.g., race, and the proportion of patients reporting at least one medical event during the study were compared between groups using the Pearson chi-square test for homogeneity of distribution. The number of days in the hospital until discharge was investigated using a two-way ANOVA. The evaluability rate, stratified by clinical site, was compared between treatment groups using the Cochran-Mantel-Haenszel test. All statistical tests were two-sided tests. The results were reported as statistically significant if $P \leq 0.05$ was obtained.

### RESULTS

### Patients

Among the participating institutions, a total of 579 patients were enrolled: 292 in the trospectomycin group and 287 in the clindamycin group. All 579 patients received study medication and were thus evaluable for safety. The two groups were comparable with regard to age, weight, height, race, and initial temperature (Table 1). Forty-nine patients from the trospectomycin group and 45 from the clindamycin group were excluded for the efficacy evaluation. The reasons for exclusion included protocol violations as well as use of concomitant antibiotic, and other foci of infection. In fact, patients receiving more than one dose of preoperative antibiotics constituted the most common reason for exclusion, followed by normal white cell count at the time of admission in the study. The total numbers of patients excluded were 28 in the trospectomycin group and 25 in the clindamycin group for the above reason. There were 4 patients in the tro-

TABLE 1. Patient characteristics: all patients

| Variable | Trospectomycin (n = 292) | Clindamycin (n = 287) |
|---|---|---|
| Mean age ± SD (years) | 26.2 ± 8.91 | 25.8 ± 8.52 |
| Weight ± SD (kg) | 76.7 ± 20.46 | 77.8 ± 17.95 |
| Height ± SD (cm) | 160.4 ± 7.03 | 160.8 ± 7.69 |
| Race (%) | | |
| Black | 51.4 | 48.3 |
| White | 17.5 | 22.3 |
| Hispanic | 28.1 | 28.2 |
| Oriental | 1.4 | 0.7 |
| Others | 1.6 | 0.5 |
| Initial temperature ± SD (°C) | 38.5 ± 0.64 | 38.5 ± 0.59 |

spectomycin group and 3 in the clindamycin group who were excluded because of their exposure to concomitant antibiotics. The concomitant use of antibiotics was primarily because of positive test for syphilis or chlamydia. Furthermore, 10 patients in each of the above groups were excluded from the study because of other foci of infection, i.e., pulmonary, ocular, or urinary tract. Seven patients in each of these two groups requested to be withdrawn from the study. The exclusion rate was not statistically different between the treatment groups. Evaluable patients and indications for inclusion are listed in Table 2.

### Outcome

The overall mean duration of the study drug therapy was similar in both groups, i.e., 4.1 ± 1.11 days. The overall success rate for the trospectomycin group was 223/243 (91.8%) compared with 214/242 (88.4%) for the clindamycin group ($P = 0.218$; Table 3).

There was a higher proportion of pretreatment isolates resistant to clindamycin than to trospectomycin. Among all the isolates from all treated patients that were tested for susceptibility to trospectomycin, the number of isolates resistant to trospectomycin was 42/1,050 (4%). In comparison, among all the isolates from all treated patients that were tested for susceptibility to clindamycin, the number of isolates resistant to clindamycin was 379/809 (47%). The most common organism isolated among the treated patients was *Enterococcus*. Of the 204 *Enterococcus* isolates that were tested for susceptibility to trospectomycin, two were resistant to trospectomycin. On the other hand, of the 185 *Enterococcus* isolates tested for susceptibility to clindamycin, 158 were resistant to clindamycin. The

TABLE 2. Indications: evaluable patients

| Indication | Trospectomycin | Clindamycin |
|---|---|---|
| Post-cesarean endometritis | 200 | 202 |
| Post-hysterectomy pelvic cellulitis | 43 | 40 |
| Total | 243 | 242 |

sensitivity for gram-positive aerobes, other than *Enterococcus* spp., was similar for trospectomycin and clindamycin. All gram-negative aerobes were resistant to clindamycin, while sensitivity to trospectomycin ranged from 0% for *Pseudomonas* to 100% for *Acinetobacter*. The sensitivity of *Escherichia coli* to trospectomycin was 93%. The sensitivity of *Bacteroides* spp. was 82% and 79% for trospectomycin and clindamycin, and for *Peptostreptococcus* it was 100% and 97%, respectively.

The overall frequency of adverse events was not significantly different between the two groups; 28% of patients in the trospectomycin group had one or more medical events compared with 28% of those treated with clindamycin. Medical events relating to the gastrointestinal system were the most frequent, with 24/292 (8%) patient complaints for trospectomycin and 29/287 (10%) for clindamycin. Of the 24 patients who reported gastrointestinal events in the trospectomycin group, the most commonly reported complaint was diarrhea (7 events) and for 29 patients in the clindamycin group, diarrhea was again the most common complaint (13 events).

### Failures

Twenty failures were reported in the trospectomycin group and 28 in the clindamycin group. All of these patients had received study medications for a minimum of 48 h. The 18 patients in the trospectomycin group who continued to have fever spikes and evidence of pelvic infection were deemed treatment failures. They subsequently responded to alternate antibiotic regimens, which included various combinations of clindamycin, gentamicin, ampicillin, cephalexin, doxycycline, and amoxicillin/calvulanic acid. One patient developed septic thrombophlebitis and recovered after heparin therapy. One patient was found to have cytomegalovirus infection and recovered spontaneously after failing study medication and combination of ampicllin, clindamycin, and gentamicin. In the

TABLE 3. Clinical success rate

| | Trospectomycin | | Clindamycin | | |
|---|---|---|---|---|---|
| | N | % | N | % | P |
| Success | | | | | |
| Total | 223/243 | 91.8 | 214/242 | 88.4 | 0.218 |
| Post-cesarean endometritis | 186/200 | 93.0 | 181/202 | 89.6 | NS |
| Post-hysterectomy pelvic cellulitis | 37/43 | 86.0 | 33/40 | 82.5 | NS |
| Failure | | | | | |
| Total | 20/243 | 8.2 | 28/242 | 11.6 | NS |
| Post-cesarean endometritis | 14/200 | 7.0 | 21/202 | 10.4 | NS |
| Post-hysterectomy pelvic cellulitis | 6/43 | 14.0 | 7/40 | 17.5 | NS |

clindamycin group, 25 patients were deemed treatment failures and were subsequently treated with different antibiotics. Three patients developed wound infection and responded to local treatment.

### DISCUSSION

Clindamycin, in combination with gentamicin, has long been considered the gold standard in treatment of obstetrical and gynecologic infection. Clindamycin is bacteriostatic, and apart from its excellent activity against anaerobic organisms, has very good activity against gram-positive aerobic organisms, including nafcillin-sensitive *Staphylococcus aureus*, and non-enterococcal streptococcus. However, the disadvantage of clindamycin is its poor activity against gram-negative aerobes. In addition, the incidence of PMC may be greater with clindamycin than with other antibiotics.

Trospectomycin is a novel broad-spectrum aminocyclitol antibiotic with a broad spectrum of activity. It is a 6′ propyl analog of spectinomycin. Trospectomycin has been shown to be effective against most aerobes, anaerobes, *Neisseria gonorrhoeae*, *Chlamydia trachomatis*, and *Ureaplasma* and *Mycoplasma* spp. In a previous open-label phase II study of trospectomycin by the sponsor in patients with chlamydia pelvic infection requiring hospitalization, 93% of patients had negative culture at the end of the therapy. In another previous phase III study by the sponsor to treat post-surgical gynecologic infection, trospectomycin was effective in 92% of patients while clindamycin was effective in 87% of patients.

Aztreonam is a first member of a class of antibiotics called monolactams. Aztreonam is a totally synthetic bactericidal antibiotic with activity against a wide spectrum of gram-negative aerobic pathogens. Activity of aztreonam against gram-negative aerobic pathogens is comparable to that of gentamicin. The sponsor of this study elected to use aztreonam.

We have shown that pelvic infection following obstetric and gynecologic procedures can be treated effectively with trospectomycin and aztreonam therapy, achieving a clinical success rate of 91.8%. The success rate for clindamycin and aztreonam was 88.4%. The difference in the success rate was not statistically significant. Trospectomycin was shown to be effective against many gram-negative aerobes, while clindamycin was resistant to all gram-negative aerobes. The addition of aztreonam in both groups, an antibiotic known to be effective against gram-negative aerobes, probably was the reason for similar efficacy in the two groups.

Even though most of the enterococci were resistant to clindamycin, the clinical cure rate was similar in two groups. This may be because enterococcus may not be the sole pathogenic organism, especially in light of the polymicrobial nature of female pelvic infections.

In summary, trospectomycin in combination with aztreonam gave a similar success rate as clindamycin and aztreonam combination in treatment of obstetric and gynecologic infections. However, broad-spectrum activity of trospectomycin warrants consideration of its use as a single agent therapy in pelvic infection following obstetric and gynecologic infection.

### REFERENCES

1. Sweet RL: Anaerobic infections of the female genital tract. Am J Obstet Gynecol 122:891, 1975.
2. Sweet RL, Ledger WJ: Puerperal infections morbidity: A two year review. Am J Obstet Gynecol 177:1903, 1973.
3. Ledger WJ, Normal J, Gee C, et al.: Bacteremia on an obstetrical gynecologic service. Am J Obstet Gynecol 121:205, 1975.

4. Thadepalli H, Gorback SL, Keith L: Anaerobic infections of the female genital tract: Bacteriologic and therapeutic aspects. Am J Obstet Gynecol 117:1034, 1973.
5. Gibbs RS: Infection after cesarean section. Clin Obstet Gynecol 28:697, 1985.
6. Stovall TG, Thorpe EM, Ling FW: Treatment of post-cesarean section endometritis with ampicillin and sulbactam or clindamycin and gentamicin. J Reprod Med 38:843, 1993.
7. Eschenbach DA, Wagner GP: Puerperal infections. Obstet Gynecol 23:1003, 1980.
8. Eschenbach DA, Buchanan TM, Pollock HM, et al.: Polymicrobial etiology of acute pelvic inflammatory diseases. N Engl J Med 293:166, 1975.
9. Duff P: Prophylactic Antibiotics for Hysterectomy. In Mead PB, Hager WD (eds): Infection Protocols for Obstetrics and Gynecology. Montrale, NJ: Medical Economics Publishing, p 277, 1995.
10. George WL, Sutter VL, Finegold SM: Antimicrobial agent-induced diarrhea bacterial disease. J Infect Dis 136:822, 1977.
11. Ledger WJ, Puttler OL: Death from pseudomembranous colitis. Obstet Gynecol 45:609, 1975.
12. Zurenko GE, Yagi BH, Vavra JJ, Wentworth BB: In vitro antibacterial activity of trospectomycin (U-63366F), novel spectinomycin analog. Antimicrob Agents Chemother 32:216, 1988.
13. Betty H, Yagi BH, Schaadt RD, Zurenko GE: The bacterial activity and post-antibiotic effect of trospectomycin. Diagn Microb Infect Dis 15:417, 1992.
14. Applebaum PC, Spangler SK, Jacobs MR: Susceptibility of 539 gram positive and gram negative anaerobes to new agents, including RP 59500, biapenem, trospectomycin and piperacillin/tazobactam. J Antimicrob Chemother 32:223, 1993.
